# Processing and Polyherbal Formulation of *Tetradium ruticarpum* (A. Juss.) Hartley: Phytochemistry, Pharmacokinetics, and Toxicity

**DOI:** 10.3389/fphar.2020.00133

**Published:** 2020-03-06

**Authors:** Qi-yuan Shan, Xia-nan Sang, Hui Hui, Qi-yang Shou, Hui-ying Fu, Min Hao, Kao-hua Liu, Qiao-yan Zhang, Gang Cao, Lu-ping Qin

**Affiliations:** ^1^ College of Pharmaceutical Science, Zhejiang Chinese Medical University, Hangzhou, China; ^2^ Afﬁliated Secondary Hospital, Zhejiang Chinese Medical University, Hangzhou, China

**Keywords:** *Tetradium ruticarpum*, processing, polyherbal formulation, compatibility, phytochemistry, pharmacokinetics, toxicity, pharmacology

## Abstract

Herbal medicine is a major part of traditional Chinese medicine (TCM), which is evolved as a system of medical practice from ancient China. The use of herbal medicine is mainly based on practice and theories and concepts rooted in ancient philosophy. In the era of evidence-based medicine, it is essential to accurately evaluate herbal remedy with standard/modern medical practice approaches. *Tetradium ruticarpum* (A. Juss.) Hartley (TR), a medicinal plant with diversify bioactive components, has been broadly used to treat pain and gastrointestinal disorders in TCM. However, TR has also been reported to have potential toxicity by long-term use or excessive doses, though the associated compounds are yet to be identified. TR is usually processed, and/or combined with other herbs in TCM formulas in order to achieve a synergistic effect or reduce its toxicity. Since processing or polyherbal formulation of TR may lead to changes in its chemical composition and contents, quality, efficacy and toxicity, comparison of TR samples before and after processing, as well as its combination with other medicines, would provide useful knowledge of bioactive compounds, efficacy and toxicity of this valuable medicinal plant. Here we reviewed the recent studies about the phytochemistry, pharmacokinetic behaviors and toxicity of TR under various processing or polyherbal formulation conditions, which would expand our understanding of mechanisms of TR’s efficacy and toxicity and be valuable for quality control in industrial manufacturing, future medicinal research, and safety and rational use of TR in TCM.

## Introduction

Traditional Chinese medicine (TCM) has been practiced in China for more than 2,500 years and herbal medicine (HM) is an indispensable part of it. HMs are plant tissues characterized by complex compositions with scant evidence on their efficacy, mode of action, and adverse reactions and the usage of HMs is mainly based on experience. In TCM practice, raw plants usually processed or prescribed with other herbs to achieve better medical efficacy (Cai et al., 2019; Jiang et al., 2019; Tian Q. et al., 2019). Therefore, accurate evaluation of the efficacy, toxicity, and rational use of HM with modern chemical and clinical approaches is critical for the development of TCM in the current era of evidence-based medicine.

**Figure 1:**
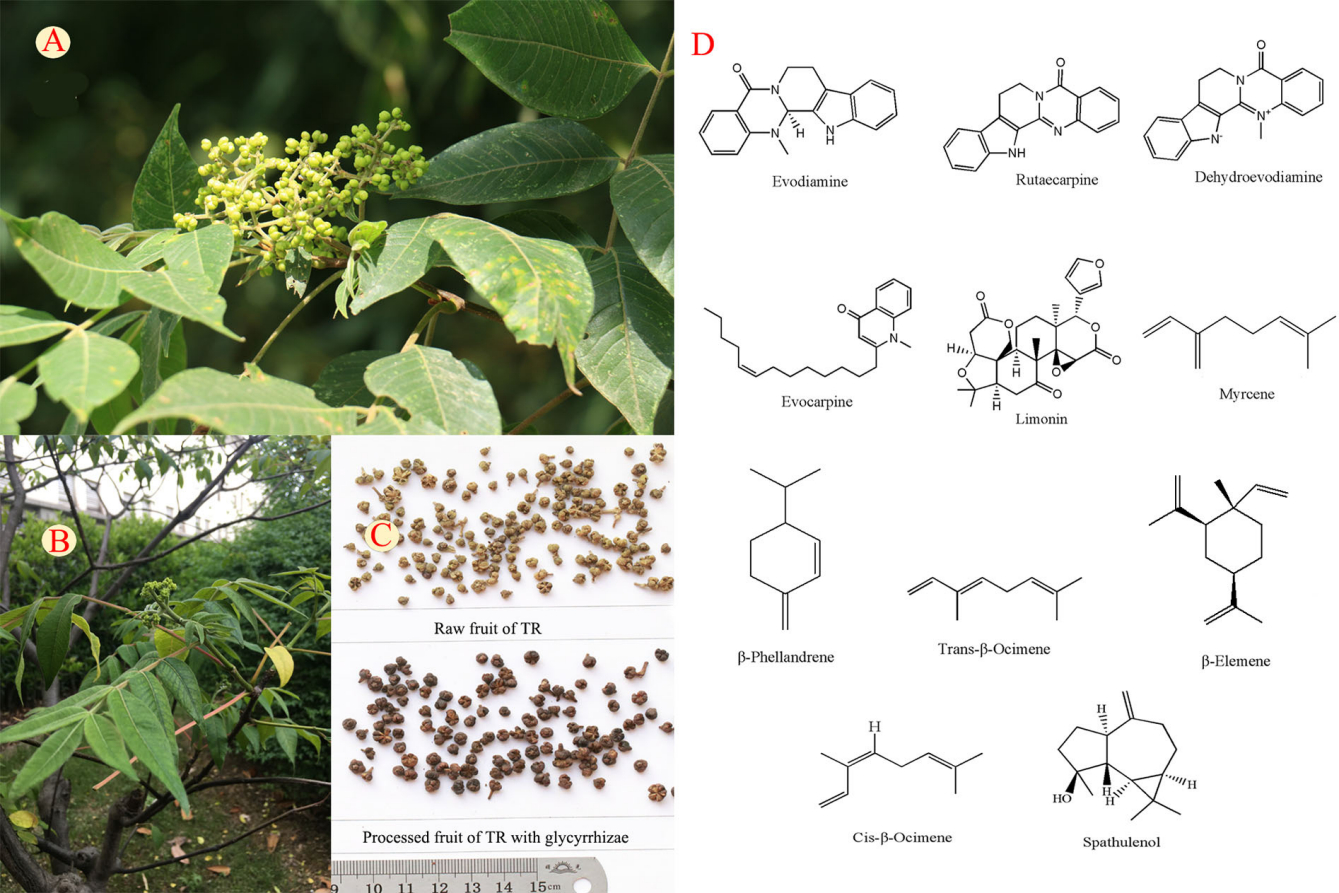
The fruit **(A)**, whole plant **(B)**, crude and processed products **(C)**, and structures of typical components **(D)** of *Tetradium ruticarpum* (A. Juss.) Hartley.

Processing is a traditional method of herbal preparations. All raw medicinal plants need to be cut, cleaned, steamed, or fried with other adjuvants before being used in TCM (Cai, 2008). Meanwhile, polyherbal formulation, or namely compatibility, is a common TCM clinical practice, where poly-herbs combined to have synergistic or balanced effects (Ekor, 2014; Ji et al., 2019; Shi et al., 2019; Wu et al., 2019). Processing or compatibility may lead to altered quality, efficacy, or safety of HM (Zhou et al., 2017). Hence, analyzing the changes of HMs before and after processing and compatibility is important for their proper and safety use. *Tetradium ruticarpum* (A. Juss.) Hartley (known hereafter as “TR”), namely *Evodiae* Fructus or *Wu zhu yu* in Chinese, is a small shrub native to temperate and tropical regions of Asia (Editorial Committee of Flora of China, 2008) and its dried nearly-ripe fruit is a frequently used HM (**Figure 1**). TR is used to treat headaches, abdominal colic, epigastric distension, dysentery, dysmenorrhea, and *post-partum* hemorrhage (Commission of Chinese Pharmacopeia, 2015), but it may have minor toxicity on excessive usage including liver toxicity in rodents, as well as vision disorders and hair loss in humans (Cai et al., 2006; Cai et al., 2014; Yin et al., 2015). Since various processing and polyherbal formulations of TR are being used clinically, TR may thus serve as a potential representative to study the chemical knowledge base of efficacy and toxicity *via* processing and compatibility approach on HMs.

Pharmacokinetics, which are usually accompanied with processing and combination of HMs, can provide clues on the modification of therapeutic effects of medicinal herbs. Moreover, the phytochemistry and toxicity of processed TR and its compatibility with other HMs can facilitate the identification of the relationships between compounds, efficacy, and toxicity. In this review, recent studies about the phytochemistry, pharmacokinetics, and toxicity of TR are summarized and discussed.

## Processing and Polyherbal Formulations

Processing and polyherbal formulation are the common practices in TCM. Accompanied with physical and/or chemical reactions, these procedures are believed to improve effects, reduce toxicity, or obtain synergistic or balanced effects. Several processing and compatibility have been reported in the use of TR. According to the 2015 Edition of *Chinese Pharmacopoeia*, stir-frying of TR with licorice water extract is the “standard” processing method, other methods include washing with hot or cold water as well as “stir-baking” with ginger juice, vinegar, salt, rice wine, black soybean water extract, or *Coptidis* Rhizoma water extract have been reported in ancient medical books and local practices. In the following sections, treatment with licorice, salt, etc. means stir-frying of TR with those compounds. Different processing methods may change the effects of TR (Xiao et al., 2017; Pei, 2018). In addition, TR is prescribed clinically with other herbs for treating various diseases, such as with *Angelica sinensis* Radix for blood deficiency, as well as mixed with *Pinellia* Rhizome and *Coptidis* Rhizoma for persistent vomiting. The composition and therapeutic effects of typical polyherbal formulations and TR preparations are summarized in **Supplementary Table S1**.

## Phytochemistry

In TCM practice, HM’s property is the basis of clinical diagnosis and treatment. As the fruit of TR is pungent and bitter, processing and compatibility will decrease its pungent aroma and bitter taste. The pungent smelling may derive from volatile oils, while the bitter taste may come from limonoids. Thus, TR’s property is closely related with its phytochemistry, which are connected with its processing or combined using application.

More than 100 compounds have been isolated or identified from the fruits of TR. Alkaloids, limonoids, volatile oils, carboxylic acids, and flavonoids are the major compounds and their typical structures are shown in **Figure 1**. Alkaloids, limonoids and some essential oils have been shown to account for anti-tumor, anti-inflammation, analgesic, and antimicrobial activities of TR (Wang X. et al., 2013; Yang, X. et al., 2013; Gavaraskar et al., 2015). The quantification changes of these components are shown in **Table 1** and the supporting identification evidences are provided in **Supplementary Figure S1**. The impacts of processing and polyherbal prescribing of TR on the major ingredients are summarized in the following sections.

**Table 1:** Contents of evodiamine, rutaecarpine, limonin, and volatile oils in *Tetradium ruticarpum* before and after processing or polyherbal formulation.

Impact factor	Processing/compatibility method	Evodiamine (%)	Rutaecarpine (%)	Limonin (%)	Volatile oils (ml/100g)	Reference
None	Raw TR	0.032 (1)	0.071 (1)	1.38 (1)	0.41 ± 5.3 (2), 0.75 ± 3.3 (3), 0.71 (4)	(1) (Zhao and Zhang, 2011) (2) (Zhang et al., 1994) (3) (Zhang et al., 2011) (4) (Chen et al., 2016)
Processing	Stir-baking with licorice juice	↓0.030 (1)	↓0.045 (1)	↓1.13 (1)	↓0.24 ± 4.8*** (2), ↓0.65 ± 3.5*** (3), ↓0.51 (4)	
	Stir-baking with salt	↑0.045 (1)	↑0.080 (1)	↓0.80 (1)	↓0.20 ± 2.9*** (2), ↓0.59 (4)	
	Stir-baking with vinegar	↓0.25 (1)	↓0.064 (1)	↓1.20 (1)	↓0.36 ± 3.7ns (2), ↓0.59 (4)	
	Stir-baking with rice wine	↑0.049 (1)	↑0.073 (1)	↓1.00 (1)	↓0.63 (4)	
	Stir-baking with *Coptidis* Rhizoma	/	/	/	↓0.65 (4)	
	Stir-baking with Ginger	↑0.044 (1)	↓0.065 (1)	↓1.29 (1)	↓0.69 (4)	
None	Raw TR extract	0.021 ± 2.23 (5) 0.014 ± 5.35 (6)	0.018 ± 0.67 (5)	/	/	(5) (Lu and Tu, 2007) (6) (Peng et al., 2012)
Polyherbal formulation	TR: *Coptidis* Rhizoma (1:6)	↑0.036 ± 2.19^###^ (5) ↑0.017 ± 7.22^##^ (6)	↑0.064 ± 0.67^###^ (5) Not detected in water extract (6)	/	/	
	TR: *Coptidis* Rhizoma (6:1)	↓0.006 ± 1.91^###^ (6)	Not detected in water extract (6)	/	/	

“↑”, “↓”, ***P < 0.001, ns, no significance, compared with contents of relative components in raw TR.

“↑”, “↓”, ^##^P < 0.01, ^###^P < 0.001, compared with contents of relative components in raw TR extract.

### Alkaloids

Alkaloids in TR are mainly composed of indole-type and quinolone-type ones. Evodiamine, rutaecarpine, and dehydroevodiamine are indole-type alkaloids, while evocarpine has the basic structure of quinolones. Some TR alkaloids have anti-inflammatory, anti-nociceptive, antibacterial, and antimalarial activities (Wang and Liang, 2004). For instance, evodiamine has been demonstrated to have multiple anti-tumor activities, while rutaecarpine offers protection against cardiovascular diseases (Yang J. et al., 2013; Yang F. et al., 2017; Tian KM. et al., 2019).

Alkaloid content varies depending on where TR was been harvested, as well as the processing procedures employed. Stir-baking with salt and wine will increase the concentrations of most of the alkaloids, while stir-baking with licorice or vinegar decrease the main alkaloids. Evodiamine and rutaecarpine could be used as two examples to study the effects of stir-baking processing on the content of alkaloids. These two compounds usually achieve higher quantity if stir-baking using rice wine or salt. When processing with ginger, the content of evodiamine increases while rutaecarpine declines (Zhao and Zhang, 2011; Zhang et al., 2019).

Alkaloid contents of TR was also affected by polyherbal formulations. The compatibility of *Coptidis* Rhizoma and TR has been evaluated. With a TR: *Coptidis* Rhizoma ratio of 1:6 in a formulation, the solubility of the main alkaloids from TR increases (and even enjoys better solubility than a TR-only decoction) while the contents of main components in *Coptidis* Rhizoma decreases (Lu and Tu, 2007; Liu et al., 2010; Peng et al., 2012; Wang L. et al., 2013).

### Limonoids

Limonoids are characterized by the basic structure of furanolactones (**Figure 1**). Since the typical structure consists of four six-membered rings and a furan ring, limonoids are classified as tetranortriterpene compounds. Limonin is a typical limonoid ingredient in TR, which has been reported to have antibacterial, antiviral, anti-tumor, and anti-obesity activities (Ono et al., 2011; Yang X. et al., 2013; Tundis et al., 2014; Zhao et al., 2019). In addition, limonin counteracts the analgesic effects of TR (Xu et al., 2019). Limonin content generally decreases after processing (Zhao and Zhang, 2011; Ma et al., 2016).

### Volatile Oils

The major volatile components in TR are terpenoids and their derivatives such as olefins, aromatic, and heterocyclic aromatic compounds. Volatile oils from TR exert analgesic, anti-inflammatory, and antibacterial effects (Huang and Sun, 2012). Most types of processing or compatible procedures can decrease volatile oil contents. A recent study showed that the volatile compounds in TR before and after processing by licorice was 0.75 and 0.65%, respectively (Zhang et al., 2011). Processing with ginger can lead to retention of most essential oils, whereas processing with vinegar and salt result in substantial loss of them (Zhang et al., 1994; Chen et al., 2016). Processing can also change the components and proportions of volatile oils. Specifically, levels of myrcene, β-phellandrene, and *trans*-ocimene are increased, whereas those of β-elemene and *cis*-ocimene are decreased, after processing using licorice (Zhang et al., 2011). TR prepared with *Coptidis* Rhizoma leads to reductions in the contents of β-phellandrene and myrcene, whereas the spathulenol level increases (Lu et al., 2003; Chen et al., 2016).

## Pharmacokinetics

Beside the chemical part explanation, the alternation of pharmacokinetic properties may be the other elucidation of TR’s changing efficacy or toxicity after processing or compatibility.

### Pharmacokinetic Parameters

Pharmacokinetic studies have mainly focused on alkaloids of TR. The concentration-time curves of evodiamine or rutaecarpine have been fitted to a one-compartment model (Luan et al., 2006; Shyr et al., 2006; Wu et al., 2007). The maximum plasma concentration (C_max_), half-life (t_1/2_), and area under the curve (AUC) for evodiamine at 500 mg/kg given orally to rats have been reported to be 49 ± 19 ng/ml, 138 ± 16.9 min, and 4.18 ± 0.74 min·μg/ml, respectively. The oral bioavailability is ~0.1% in free-moving rats (Shyr et al., 2006). The C_max_ of rutaecarpine has been reported to be 2.4 ± 3.0 ng/ml after oral administration of 40 mg/kg to rats for 18 days. Levels of rutaecarpine and evodiamine detected *in vivo* from TR extracts are much higher (22.8 ± 4.4 and 78.8 ± 23.5 ng/ml, respectively) than that of rutaecarpine alone and evodiamine alone given to rats at similar concentrations (Ding et al., 2008; Xu et al., 2012).

Processing with licorice can prolong the elimination time of alkaloid compounds in rats. The t_1/2_ of evodiamine and rutaecarpine in processed TR given at 0.4 g/kg has been reported to be 146.57 ± 38.38 and 167.10 ± 15.82 min, respectively (Bao et al., 2011). Both *in vitro* and *in vivo* studies revealed that *Coptidis* Rhizoma combined with TR leads to reduction in exposure to alkaloids from *Coptidis* Rhizoma. In the *in vitro* experiments with monolayers of Caco-2 cells under and semi-physiologic conditions, the dissolution of eight components from TR increased with the increasing proportions of *Coptidis* Rhizoma, whereas the dissolution of main alkaloids from TR increased along with the components of *Coptidis* Rhizoma decreased (Deng, 2008; Yang Y. et al., 2017). Pretreatment with TR in rats suggests that its mechanism of action involves induction of hepatic expression of uridine 5′-diphospho-glucuronosyltransferase (UGT)1A1 (Ma et al., 2011; Yan et al., 2011). Mechanism involved may be the increasing efflux of the main bioactive alkaloids of *Coptidis* Rhizoma when prescribed with TR. Since few reports have focused on the processing factors on the pharmacokinetic parameters of TR, its relationship with changes in efficacy and toxicity is yet to be explored.

### Distribution

*In vivo* studies revealed that TR processed with licorice exhibits different distribution behavior when compared with the raw TR, for the evodiamine concentration increased dramatically compared with that of the pre-processed product (Zhao, 2011). Compared with administration of *Coptidis* Rhizoma alone, combined administration with TR led to greater distribution of main alkaloids of *Coptidis* Rhizoma in the liver and less in the lungs (Liang et al., 2016). Although the findings are interesting, the mechanism of the distribution still needs further exploration.

### Metabolism

TR extracts may inhibit erythromycin activity in human liver microsomes and regulate N-demethylation levels under the presence of nicotinamide adenine dinucleotide phosphate (NADPH) *via* cytochrome P450 3A4 (CYP3A4). Rutaecarpine and limonin are the main causes of decline in residual CYP3A4 activity. Iwata et al. (2005) reported that the half-maximal inhibitory concentration (IC_50_) of rutaecarpine was > 100 and 1.4 μM before and 20 min after preincubation, respectively, and the IC_50_ of limonin was 23.5 and 1.8 μM, respectively. A competitive inhibitor of CYP3A4, ketoconazole, can attenuate inhibitory activity of limonin. CYP3A4 is one type of CYP isoform present not only in the liver, but also in the small intestine of humans. Therefore, the fruit of TR could induce drug interactions *via* CYP3A4 in the small intestine, thereby demonstrating a potential for alteration of efficacy before and after processing. Besides, according to studies using rat liver microsomes, hepatic UGT1A1 is believed to induce decreased exposure to the alkaloids of *Coptidis* Rhizoma, with the activity and expression of UGT1A1 being identified after TR pretreatment for 2 weeks (Ma et al., 2011). This action could explain why the dissolution of *Coptidis* Rhizoma declined after combination with TR. In another study, rutaecarpine was given consecutively to mice for 7 days (10, 20, and 30 mg/kg, p.o.). The transcription expressions of CYP450 genes *Cyp1a2*, *2b10*, *2e1*, *3a11*, and *4a10* were induced, as were the genes encoding hepatic transporters and phase-2 enzymes, which could provide clues of herb-drug interactions (Zhu et al., 2013).

## Pharmacology

TR is a high-frequently prescribed medicinal plant in oriental history for anti-nociceptive and gastrointestinal disorders (Yu et al., 2000; Liu et al., 2003; Yu et al., 2006), anti-cancer (Pan et al., 2012; Park et al., 2018), anti-Alzheimer's disease (Park et al., 1996; Wang et al., 2001), anti-obesity (Rebhun et al., 2015; Nie et al., 2016), cardiovascular effects (Seya et al., 2016), and anti-thrombotic effect (Sheu et al., 2000). However, pharmacological studies of TR are still in its infant stage. So far, only a few studies considered the effects of TR before and after processing (**Supplementary Table S2**). Salt and vinegar stir-baking products of TR demonstrated better analgesic activities, while licorice product show better anti-inflammatory effect (Deng and Li, 1999). Moreover, some limitations exist in early pharmacological studies, as results commonly presented in the format of original data without calculating the relative inhibition of these pharmacological activities. Furthermore, statistic analysis were mainly conducted by student’s t-test, which may not be suitable for comparisons of more than two groups. In addition, negative controls (TR before processing or polyherbal formulation) were missing in many earlier studies. Symmetrical and critical pharmacological evaluation of TR are necessary in future studies.

## Toxicity

Although HMs have long been used in history, the toxicological information and toxicity assessment of these herbal products is quite scare (Ekor, 2014). Similarly, the toxic components, mode of toxicity, prevention, and diagnose method of TR’s potential toxicity is still to be extensively investigated.

Toxicity of TR’s product before and after processing has only been reported in several studies. Volatile oils from raw and processed TR were given to mice for acute-toxicity comparisons. The median lethal dose (LD_50_) of essential oils from raw TR was 2.82 [95% confidence interval (CI) 2.48–3.21] ml/kg and the LD_50_ of processed group was 2.91 (95% CI is 2.49–3.41) ml/kg; the volatile oil content decreased by 13.33% whereas LD_50_ increased by 19.15% after processing (Zhang et al., 2011). The toxic damage in rats caused by 35 days of administration of volatile oils of TR recovered essentially after 20 days (Yin et al., 2015). *In vitro* and *in vivo* toxicity experiments have also been conducted for raw, licorice-, and salt-processed TR. The processing procedure reduced the toxicity in mice and the L02 cell model, but salt processing proved to be much safer (Zhang et al., 2018). In another study, the hepatotoxicity of TR was time- and dose-correlated with increases in levels of alanine transaminase, aspartate transaminase, lactate dehydrogenase, alkaline phosphatase, and the liver index (Zhang et al., 2017; Zhang et al., 2018).

TR extracts were also compared for their toxicity. The most tolerance dosages of whole, water and ethanol extracts of TR have been reported to be 15.6, 80, and 70.6 g·kg/day, respectively; the water extract and volatile oil from TR had analgesic activity and the effect was dose-dependent, with the safety scope of the water extract being larger than that of the volatile oil (Huang and Sun, 2012). Hepatocyte swelling, cytoplasmic-sparing neodymium, nuclear swelling, focal, or unicellular necrosis were observed in a few areas, accompanied by infiltration of inflammatory cells (Zhou et al., 2011). Genetic toxicity was not observed in the ethanol extract of TR in the micronucleus assay of bone-marrow cells in mice, whereas rutaecarpine and limonin showed some genotoxicity *in vitro* for the Ames test and CHL chromosome aberration assay (Xia et al., 2014).

Possible toxic components were speculated by several researchers. Li et al. (2017) claimed that a 50% ethanol extract was responsible for the hepatotoxicity of TR. Identification of this fraction demonstrated that nine indole alkaloids, 10 quinolone alkaloids, five triterpenoids, three flavonoids, one other alkaloid, and one organic acid were present, whereas 21 components absorbed in blood were detected. However, a study with L02 cells resulted in completely different conclusion (Wang et al., 2017).They postulated that isomers of caffeoyl gluconic acid could be the hepatotoxic substance. There is still plenty of work to be done toward TR’s toxicity, to find out the exact toxic ingredients, to clarify the toxic mechanisms, to support the quality standard for industrial manufacturing, and to facilitate rational and safety use.

## Conclusions and Future Perspectives

Herbal remedies has played, and will continue to play, an irreplaceable role in the TCM development. Processing and polyherbal formulation of HMs are common TCM clinical practices, which usually accompanied with changings of quality, efficacy, and toxicity. TR is widely used in East Asia for its analgesic, anti-inflammatory, and gastrointestinal protective effect, with various processing and compatibility practices. TR is also proved to have minor toxicity. The phytochemistry, pharmacokinetics, efficacy, and toxicity of TR have been studied under various processing or combination conditions. All the recent studies are valuable for proper processing, novel lead compound design and rational use of TR in TCM, as well as study its effective and toxic mechanisms.

Nevertheless, some knowledge gaps are yet to be filled. First, the chemical investigation *in vitro* and *in vivo* is the knowledge base for pharmacological and toxicological study, by processing or multi-herb combination, changing composition, content, or pharmacokinetic properties of these components from TR are highly connected with its efficacy or toxicity alterations. Besides, bioactive/toxic screening and separation may provide a better view on its effective or toxic ingredients, thus bringing scientific support and improvement of the current quality standard of TR and its related products, which will also bring a new insight of bioactive lead compound design. Second, the impact of processing or compatibility of TR on the toxicity evaluation and toxic mechanism is not well understood. Whether TR’s toxicity attributes to its toxic constituents, toxic metabolites, or the toxic syndromes will only observed in allergic patients, should be fully investigated. Third, safety assessments concerned with HMs in recent times are highly needed, how to prevent or diagnostic their potential toxicity is becoming more vital. Biomarker investigation technology is now emerging as a powerful tool, which may indicate the toxicity of TR at different time courses are possible solutions for safety assessment, and may serve as guarantees for prevention, diagnosis, and treatment of potential toxic HM clinical application.

## Author Contributions

Q-YSha, X-NS and HH collated documents and wrote the manuscript. L-PQ and GC contributed significantly to outline and revise the manuscript. Q-YZ helped with the phytochemistry section and manuscript revision. K-HL provided photograph of crude and processed TR. Q-YSho and H-YF helped with the consulting of pharmacokinetic and toxicity sections, respectively. MH helped with summarizing the table on compatibility application.

## Funding

This study was supported by the National Natural Science Foundation of China (81703707); Traditional Chinese Medicine Research Project, Zhejiang Province of China (2019ZA038); China Scholarship Council (201908330362).

## Conflict of Interest

The authors declare that the research was conducted in the absence of any commercial or financial relationships that could be construed as a potential conflict of interest.
