# Supplementary figures and images for: Processing and Polyherbal Formulation of Tetradium ruticarpum (A. Juss.) Hartley: Phytochemistry, Pharmacokinetics, and Toxicity
Source: Front Pharmacol. 2020 Mar 6;11:133. doi: 10.3389/fphar.2020.00133 (PMC7067890; doi:10.3389/fphar.2020.00133)

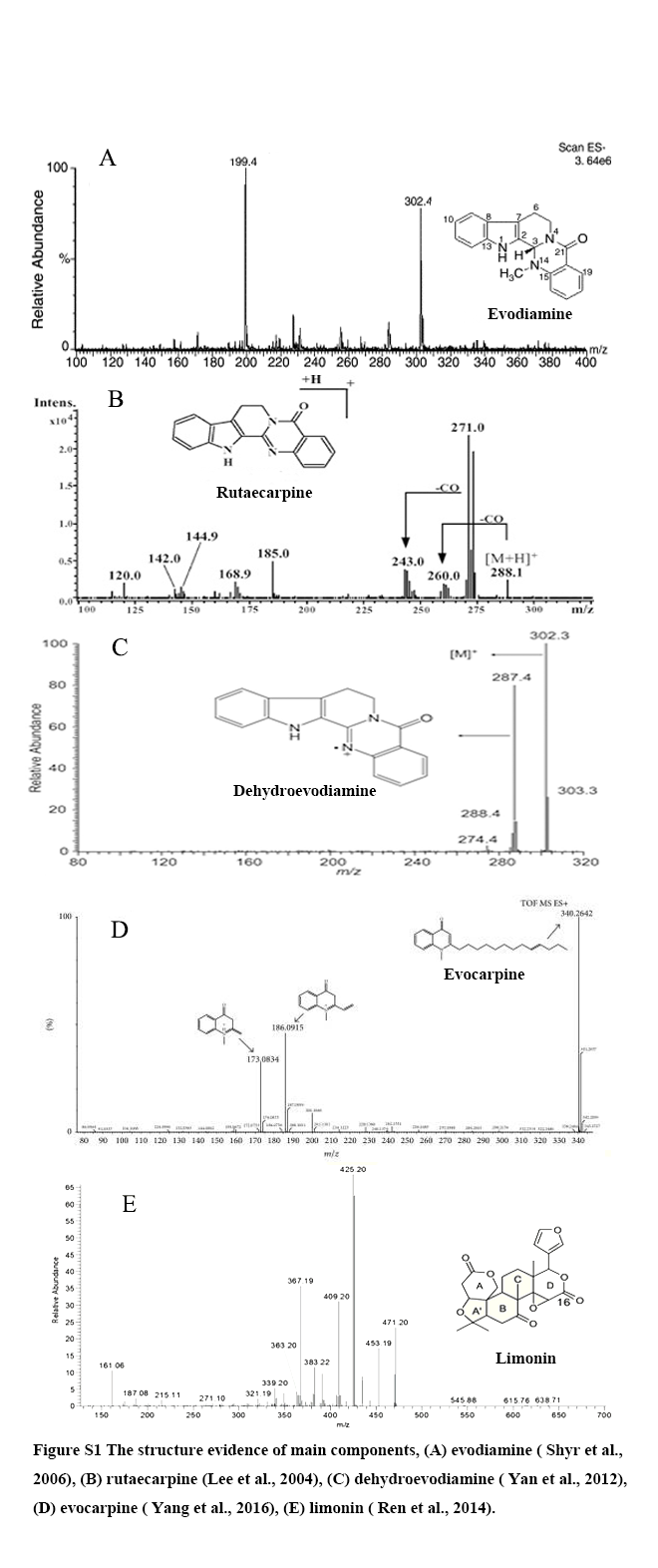

Supplement: Supplementary file 1 [file Image_1.tif]
