# Supplementary material for: Processing and Polyherbal Formulation of Tetradium ruticarpum (A. Juss.) Hartley: Phytochemistry, Pharmacokinetics, and Toxicity
Source: Front Pharmacol. 2020 Mar 6;11:133. doi: 10.3389/fphar.2020.00133 (PMC7067890; doi:10.3389/fphar.2020.00133)
Supplement: Supplementary file 2 [file Table_1.docx]

**Table S1 Typical formulas and prescriptions in traditional Chinese medicine including *Tetradium ruticarpum* (TR) fruits.**

| Formula/  Preparation name | Composition | Traditional and clinical use | References |
| --- | --- | --- | --- |
| Wu zhu yu Decoction  (also named as Goshuyu-To in Japanese) | Evodia Fructus (TR), Ginseng Radix Et Rhizoma (*Panax ginseng* C. A. Mey*.*), Zingiberis Rhizoma Recens (*Zingiber officinale* Roscore), Jujubae Fructus (*Ziziphus jujuba* Mill.) | Used for epigastrium distension, vomiting, habitual migraine, paroxysmal headache, hiccups, feeling of cold hands and feet. | *Shang Han Lun* (Treatise on Cold Pathogenic Diseases, 1066); <https://kampo.ca/herbs-formulas/>  herbs/goshuyu |
| Zuo Jin Wan | Coptidis Rhizoma (*Coptis chinensis* Franch. OR *Coptis deltoidea* C. Y. Cheng & P. K. Hsiao OR *Coptis teeta* wall.), Evodiae Fructus (TR) | Stop vomiting. | Danxi’s Mastery of Medicine, 1481; Chinese Pharmacopoeia, 2015, P. 763 |
| Wu zhu yu Decoction | Evodiae Fructus (TR), Zingiberis Rhizoma (*Zingiber offcinale* Rosc*.*) | Treating cholera. | General Records of Holy Universal Relief, 1111-1117, Vol.39 |
| Wu zhu yu Decoction | Evodiae Fructus (TR), Chaenomelis Fructus (*Chaenomeles speciosa* (Sweet) Nakai), Atractylodis Rhizoma (*Atractylodes lancea* (Thunb.) DC), Salt | Arresting vomiting, diarrhea and cramp. | Prescriptions for Universal Relief, 1406, Vol.203 |
| Wu zhu yu Decoction | Evodiae Fructus (TR), Pinelliae Rhizoma (*Pinellia ternata* (Thunb.) Makino), Aconitl Lateralis Radix Praeparata (*Aconitum carmichaelii* Debx.) | Arresting vomiting | General Records of Holy Universal Relief, 1111-1117, Vol.64 |
| Wen Jing Decoction  (also named as Unkentou in Japanese) | Evodiae Fructus (TR), Angelicae Sinensis Radix (*Angelica sinensis* (Oliv.) Diels), Chuanxiong Rhizoma (*Ligusticum striatum* DC), Paeoniae Radix Alba (*Paeonia lactiflora* Pall.), Ginseng Radix Et Rhizoma (*Panax ginseng* C. A. Mey.), etc. | Acitvating blood circulation and relieving blood stasis, amenorrhea and irregular menstration. | *Jin Gui Yao Lue* (Essential Prescriptions from the Golden Cabinet, 1066); <https://kampo.ca/herbs-formulas/>  herbs/goshuyu |
| Wu Ji Pill | Coptidis Rhizoma (*Coptis chinensis* Franch. OR *Coptis deltoidea* C. Y. Cheng & P. K. Hsiao OR *Coptis teeta* wall.), Evodiae Fructus (TR), Paeoniae Radix Alba (*Paeonia lactiflora* Pall., processed by stir frying) | Treating for burning pain in epigastric, vomiting and swallowing acid, bitter taste in mouth, abdominal pain and diarrhea. | Chinese Pharmacopoeia, 2015, P. 771 |
| Huatuo Zaizao Pill | Concentrated water-honeyed pill composed of Chuangxiong Rhizoma (*Ligusticum striatum* DC), Evodiae Fructus (TR), Borneolum Syntheticum (Borneol, C_10_H_18_O,CAS 507-70-0) et al. | Activating blood circulation, resolving phlegm, and stroke rehabilitation. | Chinese Pharmacopoeia, 2015, P. 848-849 |
| Changkang Tablet | Berberine hydrochloride (C_20_H_18_ClNO_4_, CAS 633-65-8), Aucklandiae Radix (*Aucklandia lappa* Decne.), Evodiae Fructus (TR, processed by stir-baking with *Glycyrrhizae*) | Treatment of diarrhea and dysentery. | Chinese Pharmacopoeia, 2015, P. 982-983 |
| Berberine Complex  (Fufang Huang lian su ) | Berberine hydrochloride (C_20_H_18_ClNO_4_,, CAS 633-65-8), Aucklandiae Radix (*Aucklandia lappa* Decne.), Evodiae Fructus (TR), Paeoniae Radix Alba (*Paeonia lactiflora* Pall.). | Arresting diarrhea and dysentery. Treating for acute and serve diarrhea. | Chinese Pharmacopoeia, 2015, P. 1241 |
| Dan Gui Xiang Granule | Astragali Radix (*Astragalus propinquus* Schischkin, processed by stir-frying), Cinnamomi Ramulus (*Cinnamomum cassia* (L.) J. Presl), Evodiae Fructus (TR), Cinnamomi Cortex (*Cinnamomum cassia* (L.) J. Presl), etc. | Treatment for abdominal pain, poor appetite, fullness in stomach, and chronic atrophic gastritis. | Chinese Pharmacopoeia, 2015, P. 679 |
| Ai Fu Nuan Gong Pill | Artemisiae Argyi Folium (*Artemisia argyi* H. Lév. & Vaniot, processed by stir-frying till carbonizing), Cyperi Rhizoma (C*yperus rotundus* L., processed with vinegar), Evodiae Fructus (TR, processed by stir-baking with *Glycyrrhizae*), etc. | Treating for irregular menstruation and dysmenorrhea. | Chinese Pharmacopoeia, 2015, P. 758-759 |
