# Supplementary material for: Processing and Polyherbal Formulation of Tetradium ruticarpum (A. Juss.) Hartley: Phytochemistry, Pharmacokinetics, and Toxicity
Source: Front Pharmacol. 2020 Mar 6;11:133. doi: 10.3389/fphar.2020.00133 (PMC7067890; doi:10.3389/fphar.2020.00133)
Supplement: Supplementary file 3 [file Table_2.docx]

**Table S2.** The pharmacological effects of *Tetradium ruticarpum* before and after processing or polyherbal formulations*.*

| Impact factor | Processing method | Pharmacological activities | | | | Reference |
| --- | --- | --- | --- | --- | --- | --- |
|  |  | Analgesic | | Anti-inflammatory | Anti-diarrhea |  |
|  |  | Hot plate test in mice at 30 min (Analgesic inhibition %) | Acetic acid-induced writhing in mice at 20 min (Analgesic inhibition %) | Egg yolk - induced paw edema in mice at 1 h (Paw edema%). | Folium Sennae induced diarrhea mice model (wet fecal pellet, count/24 h) |  |
| None | None (distilled water) | 2.5 | - | 283.3 | 5.6 ± 1.03 | ([Deng and Li, 1999](#_ENREF_9)) |
| None | Crude product | 36.9 | 26.8 | 130.8 | 1.8 ± 1.26 |  |
| Processing | Stir - baking with licorice juice | 64.5 | 39.3 | 58.3 | 2.0 ± 1.10 |  |
|  | Stir - baking with salt | 95.5 | 56.8 | 161.5 | 2.2 ± 1.47 |  |
|  | Stir - baking with vinegar | 60.4 | 48.1 | 192.3 | 2.5 ± 1.05 |  |
